# Supplementary material for: PredIL13: Stacking a variety of machine and deep learning methods with ESM-2 language model for identifying IL13-inducing peptides
Source: PLoS One. 2024 Aug 22;19(8):e0309078. doi: 10.1371/journal.pone.0309078 (PMC11340954; doi:10.1371/journal.pone.0309078)
Supplement: S1 Fig — (A) LGBM; (B) XGB; (C) RF; (D) SVM; (E) NB (F) KNN. (PDF) [file pone.0309078.s001.pdf]

Supplemental information

(A) LGBM

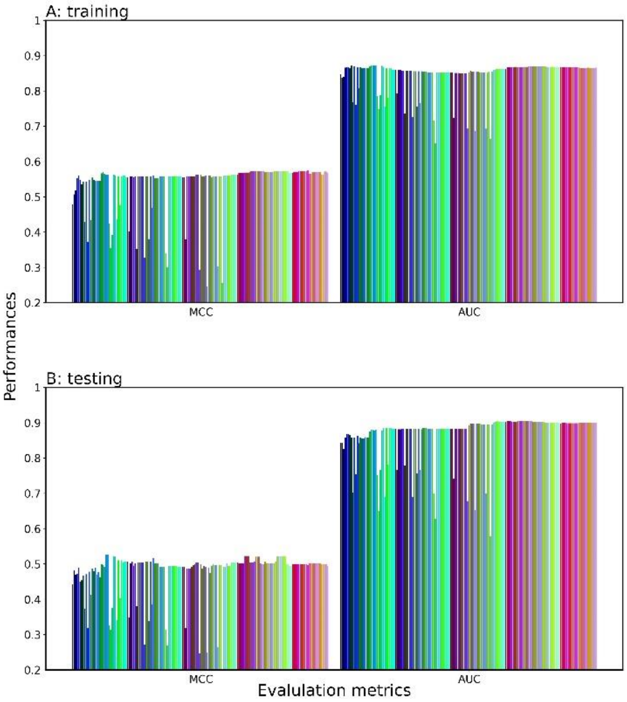

(B) XGB

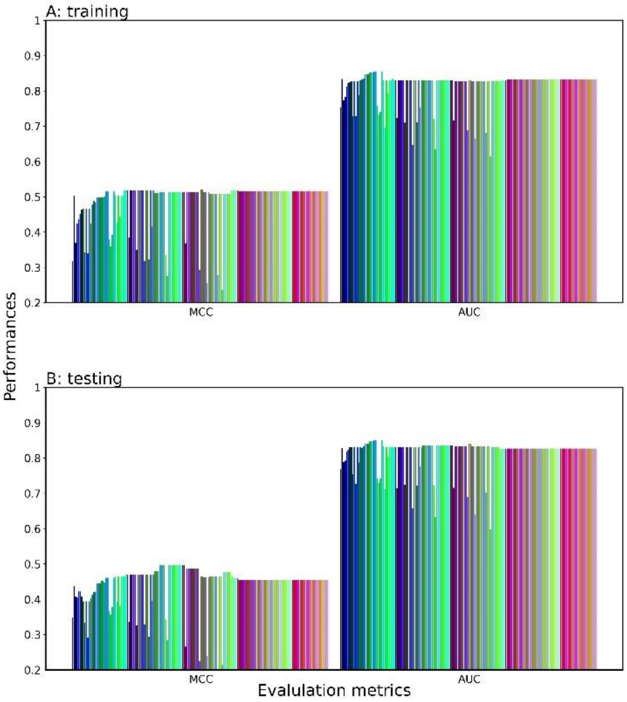

(C) RF

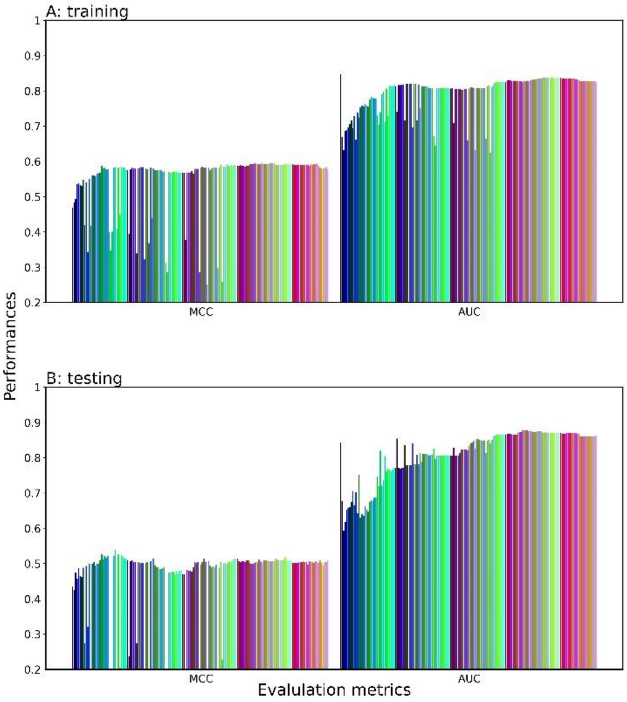

(D) SVM

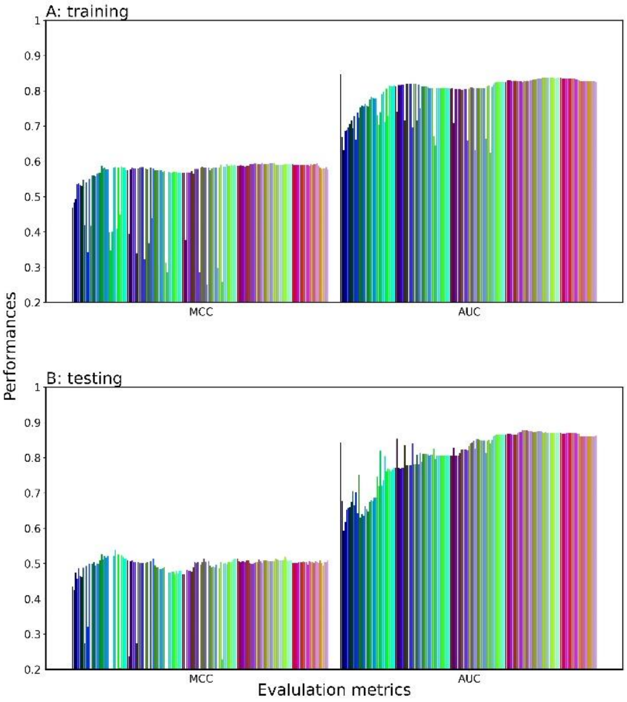

(E) NB

(F) KNN

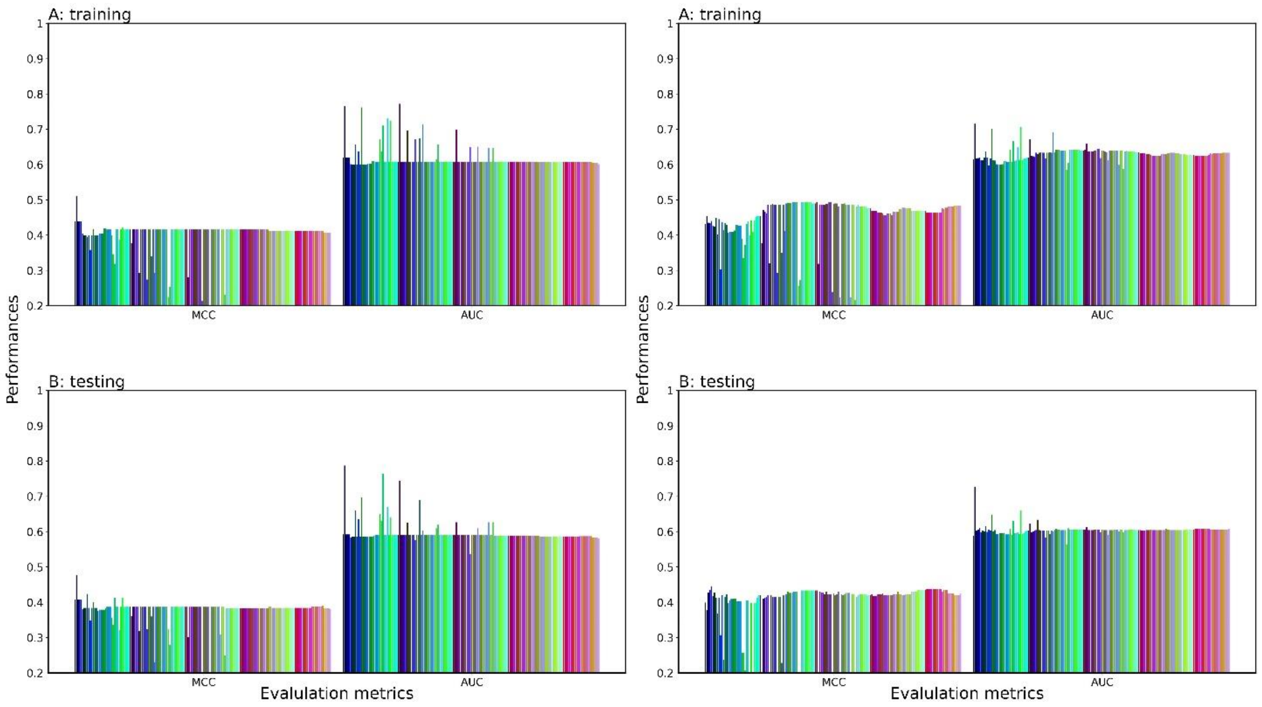

S1 Fig. Prediction performance of six meta-classifiers built by the SAAUC method on the validation and test datasets. (A) LGBM; (B) XGB; (C) RF; (D) SVM; (E) NB (F) KNN.
