# Supplementary material for: PredIL13: Stacking a variety of machine and deep learning methods with ESM-2 language model for identifying IL13-inducing peptides
Source: PLoS One. 2024 Aug 22;19(8):e0309078. doi: 10.1371/journal.pone.0309078 (PMC11340954; doi:10.1371/journal.pone.0309078)
Supplement: S2 Fig — (PDF) [file pone.0309078.s002.pdf]

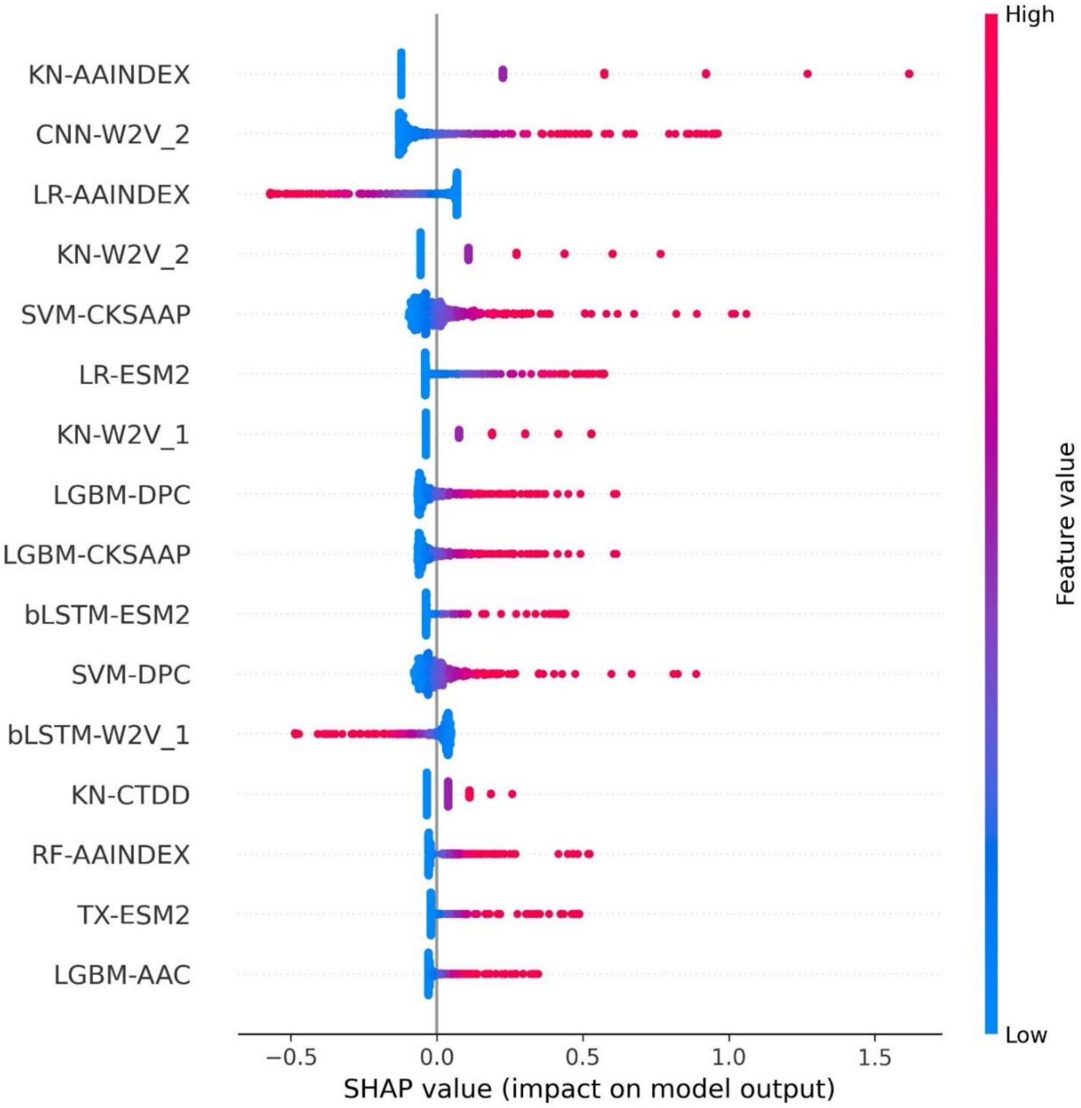

S2 Fig. SHAP analysis of the SDIWC-stacked classifier consisting of top 72 single-feature models. LR is employed to stack the 72 models with ACC > 0.902 on the training dataset. SHapley Additive exPlanations (SHAP) values assign each single-feature model (feature) a value that represents its contribution to the prediction for the individual samples. Positive SHAP values indicate the feature that increases the predicted probability towards the positive class, while negative values indicate the features that decreases it. We conducted SHAP analysis to the binary classification of PredIL13. We sorted the single-feature models according to their feature values and displayed the top 16 model (S2 Fig.). While the three deep learning methods (CNN, TX, and bLSTM) and KN were not included in the AWCLR-selected top 16 models (Table 2), the six single-feature models (LGBM-AAC, LGBM-CKSAAP, LGBM-DPC, LR-ESM-2, SVM-DPC, and SVM-CKSAAP) were included in the AWCLR-selected top 16. In addition, all the encoding methods of SHAP-selected top 16 models were consistent with those of the AWCLR-selected top 16 ones. The SHAP-selected single-feature models were rather consistent with the AWCLR-selected models.
